# A Novel Fault Feature Recognition Method for Time-Varying Signals and Its Application to Planetary Gearbox Fault Diagnosis under Variable Speed Conditions

**DOI:** 10.3390/s19143154

**Published:** 2019-07-17

**Authors:** Yong Lv, Bingqi Pan, Cancan Yi, Yubo Ma

**Affiliations:** 1 The Key Laboratory of Metallurgical Equipment and Control of Education Ministry, Wuhan University of Science and Technology, Wuhan 430081, China; 2 Hubei Key Laboratory of Mechanical Transmission and Manufacturing Engineering, Wuhan University of Science and Technology, Wuhan 430081, China

**Keywords:** time-varying signal, time-frequency analysis, multi-synchrosqueezing transform, fault diagnosis, planetary gearbox

## Abstract

The existing time-frequency analysis (TFA) methods mainly highlight the time-frequency ridges of the interested components by optimizing the time-frequency plane to facilitate the extraction of the relevant components. Generalized demodulation (GD), order tracking (OT), and other methods are generally used in conjunction with the TFA methods to realize the transition from a time-varying signal to a stationary signal, and finally identify the fault feature through a time-frequency plane. Generally, it is necessary to clarify the accuracy of the estimated components such as the rotational frequency or the fault characteristic frequency (FCF) during the operation of the GD or OT methods. Unfortunately, it is not only difficult to extract and locate rotational frequency or FCF, but also complicated in the whole estimation process. In this paper, a simple yet readable method is proposed to reveal the fault feature of time-varying signals. First, the method only needs to extract an arbitrary instantaneous frequency (IF). This is different from the GD method which needs to estimate and locate all phase functions. Then, it converts all variable frequency curves into corresponding lines parallel to the frequency axis based on the extracted IF to determine the proportional relationship between the components. Finally, to further improve the readability of the final results, we reduce the dimension of the transformed time-frequency representation to generate a two-dimensional (2D) energy-frequency map with high resolution and the same proportion. Subsequently, the performance is validated by simulated and experimental data.

## 1. Introduction

With the increasingly serious energy crisis and environmental pollution, as a green renewable energy, wind energy has attracted more and more attention throughout the world. As the main transmission component of wind turbines, planetary gearboxes are prone to failure due to long-term impact and alternating loads [1,2]. Therefore, accurate identification of gearbox fault characteristics is the prerequisite for fault diagnosis and health management of mechanical equipment [3,4].

Under the condition of variable speed, the signal of planetary gearbox has typical aperiodicity and non-stationarity. Undoubtedly, directly obtaining the fault characteristic frequencies of non-stationary signals by using the traditional Fourier spectrum analysis such fast Fourier transform is almost impossible [5,6]. Due to the aperiodic characteristics of non-stationary signals, the energy of the characteristic frequency is dispersed on the frequency axis, which is difficult to highlight the energy of a certain characteristic frequency on spectrum.

Time-frequency analysis (TFA) has always been a powerful tool for dealing with a non-stationary signal [7,8,9,10]. In recent decades, many TFA methods based on the short-time Fourier transform (STFT) and the continuous wavelet transform (CWT) have been proposed by scholars and applied in the field of mechanical fault diagnosis [11,12,13,14,15]. However, although the linear time-frequency analysis methods represented by the STFT and the CWT can be used to analyze non-stationary signals, time-frequency distribution with perfect time and frequency resolution cannot be obtained simultaneously, which is limited by the Heisenberg uncertainty principle [16,17]. Similarly, although the bilinear time-frequency analysis method represented by the Wigner–Ville distribution has effectively improved the resolution of time-frequency distribution, the analysis results of this method are prone to interference by cross terms and not suitable for analyzing multicomponent signals [17,18]. Moreover, due to the typical modulation characteristics of time-varying signals, energy divergence and mode aliasing are easy to occur when using traditional TFAs, which directly blur the time-frequency representation (TFR) and make it difficult to extract the accurate component information [19].

In order to achieve the concentrated time-frequency expression, I. Daubechies proposed the synchrosqueezed transform (SST), which is based on time-frequency coefficient rearrangement technology and has been applied in many fields, such as medicine, geophysics, and audio [20]. The SST is a special redistribution algorithm, which corresponds to a reallocation operator to improve the time-frequency resolution. In SST, the time-frequency coefficient is reallocated only on the frequency axis, which not only simplifies the distribution process but also keeps contact with the original parameters. Thakur et al. proposed the SST based on the STFT and this method had been successfully applied to ECG signals [21]. Presently, the SST has been widely used in the fault diagnosis of key parts of mechanical equipment [22,23,24,25]. However, the frequency reallocation operator in SST cannot provide an unbiased estimation for real-time accuracy, which makes it only sharpen the slowly changing signals and cannot meet the requirement of the high time-frequency resolution. For this reason, Pham et al. put forward the high-order synchrosqueezing transform (HSST) [26]. The HSST redefines the reallocation operator based on the high-order amplitude and phase approximation by using the Taylor expansion, which further improves the accuracy of the extracted instantaneous frequency (IF). Theoretically, the HSST is based on sharpening fast changing signals, however, the ability of HSST is related to the degree of the Taylor expansion. With the improvement of the order of the Taylor expansion, the corresponding expression of the algorithm will become complex. To make matters worse, the computation numbers of the STFT increase in the form of a square of the order, which greatly increases the calculation cost.

Recently, the proposed multi-synchrosqueezing transform (MSST) method has a better performance in terms of further sharpening the TFR than the HSST [27]. The MSST method introduces a progressive idea into the SST, and therefore makes it possible to estimate IF more accurately through repeated estimation of the reallocation operator, since this method only optimizes the reallocation operator and the whole process only needs to calculate the STFT once. With the increase of iterations, the MSST method can obtain time-frequency representation with higher resolution than the HSST. Although the HSST and MSST methods achieve further energy concentration, it is still difficult to intuitively obtain interesting information from the TFR in the background noise, for the same theoretical basis.

Generalized demodulation (GD) [28,29,30] transforms the IF of any time-varying signal into a constant frequency, and each component signal is extracted by means of bandpass filtering, which largely makes up for the deficiency of time-frequency analysis methods based on the STFT and the CWT in signal processing. However, GD needs to estimate the phase function of all interested components, which is very complicated in practical use. Similarly, order tracking (OT) [31,32] is a powerful tool commonly used in non-stationary signal processing. First, this approach estimates the shaft frequency accurately, then, realizes the signal stabilization, and finally, presents in the form of an order spectrum. However, the angle domain resampling method is adopted in OT, which inevitably leads to the problems of missing values and interpolation. In addition, due to the lack of noise reduction operation, the background noise is still retained in the order spectrum.

In this paper, a new method of fault feature recognition for time-varying signals is proposed. The main purpose of this method is to simplify the analysis process of GD and present more explicit and readable results. For this reason, we focus on the following:

1. Can the process of estimating and locating all phase functions in GD with only one instantaneous frequency be simplified and achieve similar smooth transformation?
2. Can the process be further simplified? For example, can the instantaneous frequency that needs to be estimated be arbitrary?
3. Can more intuitive and readable results on the basis of TFR be presented?

In order to realize this idea, we mainly improve the following algorithms:

1. To obtain stable high resolution TFR, an improved multi-synchrosqueezing transform (IMSST) algorithm based on MSST is proposed. According to the intermediate frequency extracted in the first step, the time-varying full frequency can be directly converted into stable full frequency.
2. To transform the instantaneous frequency ridges into a series of lines parallel to the frequency axis, we improve the instantaneous frequency estimation operator based on the MSST algorithm, so that we achieve the result which is similar to GD using only an arbitrary extracted IF.
3. To present more intuitive and readable results, we propose a simple data dimension reduction method, which generates a more readable two-dimensional (2D) energy-frequency diagram.

This paper contributes to the following three major aspects:

1. A three-step model is used to enhance readability of the final results.
2. The proposed method is validated in multicomponent and planetary gearbox simulation signals in the form of increasing signal complexity.
3. The proposed method is applied to diagnose the planetary gearbox fault under a time-varying condition and directly recognize the fault type from the 2D energy-frequency map without using any other method.

## 2. Theoretical Description

According to the previous ideas, the proposed method is mainly divided into three steps: firstly, extract an arbitrary instantaneous frequency; then, obtain steady and high resolution TFR from time-varying signals; finally, produce a 2D energy-frequency map. Therefore, in this section, we will introduce the relevant theories of these three steps in turn.

### 2.1. Extraction Algorithm of IF

At present, the peak searching algorithm is the most common algorithm in IF extraction. It obtains the IF of interested components by searching the peak of time-frequency ridge energy. However, due to the interference of modulation phenomenon and strong noise in measured signals, it is generally difficult to extract high accuracy time-frequency ridges directly.

In Ref. [33], G. Yu proposed a general linear chirplet transform (GLCT). He used a time-varying demodulation operator to produce a rotational effect on the time-frequency plane, so as to solve the problem of energy divergence caused by modulation. The GLCT constructs discrete time-varying demodulation operators by defining rotating degree α which is written as follows:

(1) $$\{\begin{array}{l} \alpha =\arctan(\frac{T}{f_{s}}c),\;\alpha \in (-\frac{\pi }{2},\frac{\pi }{2})\\ \alpha =-\frac{\pi }{2}+\frac{\pi }{N+1},-\frac{\pi }{2}+\frac{2\pi }{N+1},\cdots ,-\frac{\pi }{2}+\frac{N\pi }{N+1},(N=1,2,\cdots ) \end{array}$$

where, T is sampling time and $f_{s}$ is sampling frequency. N denotes the number of discrete time-varying demodulation operator c. From Equation (1), the rotating degree α is equally divided into *N* parts. However, considering the difference of the instantaneous change trend of general time-varying signals, it is difficult to completely represent the change trend of the time-frequency curve only through the division of equal intervals. 

In this paper, we abandon the above estimation form and reconstruct the time-varying demodulation operator directly by estimating the trend of the instantaneous frequency. In this way, the energy concentration of the instantaneous frequency curve is maintained to the greatest extent.

An IF extraction algorithm based on time-varying demodulation and the STFT (TDSTFT) is proposed, which combines the idea of time-varying demodulation and the peak searching algorithm. It repeatedly estimates the time-varying demodulation operator through the changing trend of IF to optimize the TFR, and then extracts ridge line using the peak searching algorithm. The following is a detailed theoretical introduction.

An AM-FM signal is defined as:

(2) $$s(t)=A(t)e^{i\Phi (t)}=A(t)e^{i\int \varphi (t)dt}$$

where, *A*(*t*) and Φ(*t*) are their instantaneous amplitude and instantaneous phase, respectively. And $\varphi (t)=\Phi^{\prime }(t)$ is regarded as the ideal IF of the analytical signal. In a short time u, the IF of signal s(t) can be approximately regarded as a linear equation, which is expressed by the first-order Taylor expansion:

(3) φ(u)=φ(t)+φ(t)(u−t)

Then, the corresponding STFT is expanded into the following form:

(4) $$\begin{array}{ll} \left|S(\varphi (t)\right| & =\left|\int_{-\infty }^{+\infty }g(u-t)s(u)e^{-i\varphi (t)u}du\right|\\ & =\left|\int_{-\infty }^{+\infty }g(u-t)A(u)e^{i[\varphi (t)u+\varphi (t){\left(u-t\right)}^{2}/2]}e^{-i\varphi (t)u}du\right|\\ & =\left|\int_{-\infty }^{+\infty }e^{i\varphi (t){\left(u-t\right)}^{2}/2}g(u-t)A(u)e^{i\varphi (t)u}e^{-i\varphi (t)u}du\right|\\ & \leq \left|\int_{-\infty }^{+\infty }g(u-t)A(u)du\right| \end{array}$$

where, g(t) is a window function. Here we choose the Gauss window, whose expression is written as:

(5) $$g(t)=\frac{1}{\sqrt{2\pi }\sigma }e^{-\frac{1}{2\sigma^{2}}t^{2}}$$

According to Equation (4), the signal is modulated and the energy of STFT diverges due to the presence of $e^{i\varphi (t){\left(u-t\right)}^{2}/2}$. Therefore, we introduce the time-varying demodulation operator $e^{i\tilde{c}(t){\left(u-t\right)}^{2}/2}$ to eliminate the influence of $e^{i\varphi (t){\left(u-t\right)}^{2}/2}$, which makes the TFR energy more concentrated. After introducing the time-varying demodulation operator, the STFT is rewritten as:

(6) $$TDSTFT=\int_{-\infty }^{+\infty }g(u-t)s(u)e^{-i\varphi (t)u}e^{-i\tilde{c}(t){\left(u-t\right)}^{2}/2}du$$

Because the $\tilde{c}(t)$ is unknown, here, we adopt the approximation strategy and achieve the accuracy allowed by the algorithm through the change trend of the approximate IF step by step. Considering the uniformity of energy concentration, this paper directly uses the change trend of instantaneous frequency to express $\tilde{c}(t)$.

(7) $$\tilde{c}(t)=\varphi^{\prime }(t)$$

The specific process of the whole extraction algorithm (Algorithm 1) is as follows:

**Algorithm 1** TDSTFT
**Input:**
s(t)

**Output:**
$\tilde{c}(t),\;TDSTFT$
**1:** **Initialize:**$\tilde{c}(t)=0$; the largest number of iterations: n=1,00; convergence threshold ε.**2:** **Calculate:**    $TDSTFT=\int_{-\infty }^{+\infty }g(u-t)s(u)e^{-i\varphi (t)u}e^{-i\tilde{c}(t){\left(u-t\right)}^{2}/2}du$;    $L_{t}=length\left(s(t)\right)$;    P=peak−searching(TDSTFT);    I(t)=polyfit(P);    I(t)=polyfit(P);    $I^{\prime }(t)=diff(I(t))$;**3:** **Iteration:**    **for**
j=1:n;     $I_{i}(t)=I(t)$;     $\tilde{c}(t)=I^{\prime }(t)$;     $TDSTFT=\int_{-\infty }^{+\infty }g(u-t)s(u)e^{-i\varphi (t)u}e^{-i\tilde{c}(t){\left(u-t\right)}^{2}/2}du$;     P=peak−searching(TDSTFT);     I(t)=polyfit(P);     $I^{\prime }(t)=diff(I(t))$;     $\xi =\frac{1}{L_{t}}\int_{0}^{L_{t}}\left|I(t)-I_{i}(t)\right|$;     **if**
ξ<ε;      **breake;**     **end**    **end**

The IF of a component can be obtained more accurately after the above steps. The following is an example to illustrate the precision of the IF extraction algorithm.

**Figure 1:**
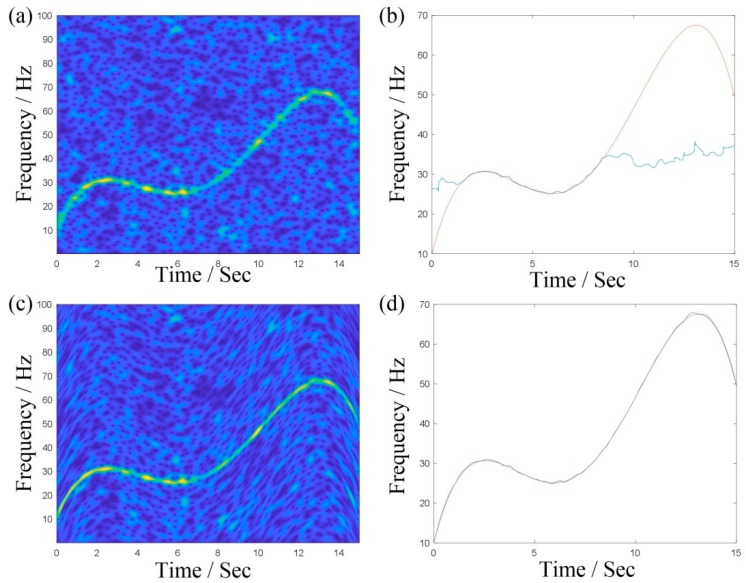
(**a**,**b**) are the TFR and extracted instantaneous frequency (IF) obtained using short-time Fourier transform (STFT); (**c**,**d**) are the TFR and extracted IF obtained using time-varying demodulation and the STFT (TDSTFT).

We use a numerical example to illustrate the effectiveness of this method and the simulated signal is $s(t)=\sin\left[2\pi \left(10t+10t^{2}-\frac{19}{9}t^{3}+\frac{9}{50}t^{4}-\frac{1}{200}t^{5}\right)\right]+n(t)$, where n(t) is −2 dB while Gaussian noise and the sampling frequency is set to 200 Hz. Figure 1a,c shows the TFRs by STFT and TDSTFT after four iterations, respectively. The energy of TFR, as shown in Figure 1c, is more concentrated. Figure 1b,d shows the extracted IFs obtained from Figure 1a,c, respectively, using the peak searching algorithm (where, the blue line is the extracted IF and the red line is the real value). Through the comparative analysis, it is illustrated that the IF extracted from graph d in Figure 1 is obviously closer to the true value, which also proves the effectiveness of the proposed method.

### 2.2. The TFRs by IMSST

According to the expression of time-varying signals in Equation (2), a standard STFT is written as follows:

(8) $$V_{f}^{g}(t,\eta )=\int s(\tau )g^{*}(\tau -t)e^{-i\eta (\tau -t)}d\tau$$

where, $g^{*}(t)$ is the complex common of g(t).

Then, the SST based on the STFT is expressed as:

(9) $$Ts(t,\omega )=\frac{1}{g^{*}\left(0\right)}\int_{\left\{\eta ,\left|V_{f}^{g}(t,\eta )\right|>\gamma \right\}}^{\left(t,\eta \right)\to \left(t,\omega \right)}V_{f}^{g}(t,\eta )\delta (\omega -{\hat{\omega }}_{f}(t,\eta ))d\eta$$

where, γ is a very small threshold to prevent excessive error when $V_{f}^{g}(t,\eta )=0$. And δ function is defined as:

(10) $$\delta (\omega -\omega_{f})=\{\begin{array}{ll} 1, & if\;\omega =\omega_{f}\\ 0, & f\;\omega \neq \omega_{f} \end{array}$$

In Equation (9), $\omega_{f}(t,\eta )$ represents the IF estimation of signals on t and η. Since the phase after STFT is not affected by the window function g(t), we use $V_{f}^{g}(t,\eta )$ to estimate the IF:

(11) $$\omega_{f}(t,\eta )=\frac{\partial }{\partial_{t}}\arg\left[V_{f}^{g}(t,\eta )\right]=\frac{\partial_{t}V_{f}^{g}(t,\eta )}{iV_{f}^{g}(t,\eta )}$$

where, arg[A] denotes the angle of the complex A. Considering that $\omega_{f}(t,\eta )$ is a complex number, we only take the real part as the estimation of IF.

(12) ω^f(t,η)=Re{ωf(t,η)}=Re{∂tVfg(t,η)iVfg(t,η)}

Although SST cannot deal with strong time-varying signals well, TFR with high concentration can also be obtained by continuously utilizing SST concentrating energy. This is the core idea of MSST method.

(13) $$\begin{array}{c} Ts^{\left[2\right]}(t,\omega )=\int Ts^{\left[1\right]}(t,\eta )\delta (\omega -{\hat{\omega }}_{f}(t,\eta ))d\eta \\ Ts^{\left[3\right]}(t,\omega )=\int Ts^{\left[2\right]}(t,\eta )\delta (\omega -{\hat{\omega }}_{f}(t,\eta ))d\eta \\ \vdots \\ Ts^{\left[N\right]}(t,\omega )=\int Ts^{\left[N-1\right]}(t,\eta )\delta (\omega -{\hat{\omega }}_{f}(t,\eta ))d\eta \end{array}$$

when N=2,

(14) $$\begin{array}{ll} Ts^{\left[2\right]}(t,\omega ) & =\int Ts^{\left[1\right]}(t,\xi )\delta (\xi -{\hat{\omega }}_{f}(t,\xi ))d\xi \\ & =\iint V_{f}^{g}(t,\eta )\delta (\omega -{\hat{\omega }}_{f}(t,\eta ))d\eta \delta (\omega -{\hat{\omega }}_{f}(t,\xi ))d\xi \\ & =\int V_{f}^{g}(t,\eta )\int \delta (\omega -{\hat{\omega }}_{f}(t,\eta ))\delta (\omega -{\hat{\omega }}_{f}(t,\xi ))d\xi d\eta \\ & =\int V_{f}^{g}(t,\eta )\delta (\omega -{\hat{\omega }}_{f}(t,{\hat{\omega }}_{f}(t,\eta )))d\eta \end{array}$$

After the second iteration, the original time-frequency plane is changed from (t,η) to $\left(t,{\hat{\omega }}_{f}(t,{\hat{\omega }}_{f}(t,\eta ))\right)$. 

In a short time, the phase Φ(τ) of the original signal s(τ) is expanded as:

(15) $$\Phi (\tau )=\Phi (t)+\Phi^{\prime }(t)(\tau -t)+\frac{1}{2}\Phi^{\prime \prime }(t){\left(\tau -t\right)}^{2}$$

The purpose of (15) is to simplify the STFT coefficient $V_{f}^{g}(t,\eta )$ and $V_{f}^{g}(t,\eta )$ is rewritten as follows:

(16) $$\begin{array}{ll} V_{f}^{g}(t,\eta ) & =\int A(\tau )e^{i[\Phi (t)+\Phi^{\prime }(t)(\tau -t)+\frac{1}{2}\Phi^{\prime \prime }(t){\left(\tau -t\right)}^{2}]}g^{*}(\tau -t)e^{-i\eta (\tau -t)}d\tau \\ & =A(\tau )e^{i\Phi (t)}\frac{1}{\sqrt{1-i\Phi^{\prime \prime }(t)}}e^{-\frac{{\left(\eta -\Phi^{\prime }(t)\right)}^{2}}{2(1-i\Phi^{\prime \prime }(t))}} \end{array}$$

Similarly, the IF estimation is expressed as:

(17) ω^f(t,η)=Re{∂tVfg(t,η)iVfg(t,η)}=Φ′(t)+(Φ″(t))21+(Φ″(t))2(η−Φ′(t))

When N=2,

(18) $$\begin{array}{ll} {\hat{\omega }}_{f}(t,{\hat{\omega }}_{f}(t,\eta )) & =\Phi^{\prime }(t)+\frac{{\left(\Phi^{\prime \prime }(t)\right)}^{2}}{1+{\left(\Phi^{\prime \prime }(t)\right)}^{2}}({\hat{\omega }}_{f}(t,\eta )-\Phi^{\prime }(t))\\ & =\Phi^{\prime }(t)+{\left[\frac{{\left(\Phi^{\prime \prime }(t)\right)}^{2}}{1+{\left(\Phi^{\prime \prime }(t)\right)}^{2}}\right]}^{2}\left(\eta -\Phi^{\prime }(t)\right) \end{array}$$

When N=3,

(19) $$\begin{array}{ll} {\hat{\omega }}_{f}^{\left[3\right]}(t,\eta ) & ={\hat{\omega }}_{f}(t,{\hat{\omega }}_{f}(t,{\hat{\omega }}_{f}(t,\eta )))\\ & =\Phi^{\prime }(t)+{\left[\frac{{\left(\Phi^{\prime \prime }(t)\right)}^{2}}{1+{\left(\Phi^{\prime \prime }(t)\right)}^{2}}\right]}^{2}\left({\hat{\omega }}_{f}(t,\eta )-\Phi^{\prime }(t)\right)\\ & =\Phi^{\prime }(t)+{\left[\frac{{\left(\Phi^{\prime \prime }(t)\right)}^{2}}{1+{\left(\Phi^{\prime \prime }(t)\right)}^{2}}\right]}^{2}\left[\frac{{\left(\Phi^{\prime \prime }(t)\right)}^{2}}{1+{\left(\Phi^{\prime \prime }(t)\right)}^{2}}\left(\eta -\Phi^{\prime }(t)\right)\right]\\ & =\Phi^{\prime }(t)+{\left[\frac{{\left(\Phi^{\prime \prime }(t)\right)}^{2}}{1+{\left(\Phi^{\prime \prime }(t)\right)}^{2}}\right]}^{3}\left(\eta -\Phi^{\prime }(t)\right) \end{array}$$

In order to facilitate the writing, here, we use ${\hat{\omega }}_{f}^{\left[3\right]}(t,\eta )$ instead of ${\hat{\omega }}_{f}(t,{\hat{\omega }}_{f}(t,{\hat{\omega }}_{f}(t,\eta )))$. Similarly, the estimation of IF ${\hat{\omega }}_{f}(t,{\hat{\omega }}_{f}(t,{\hat{\omega }}_{f}(t,{\hat{\omega }}_{f}(t,\cdots ))))$ after the *N*th iteration is written as ${\hat{\omega }}_{f}^{\left[N\right]}(t,\eta )$. According to the law, it is not difficult to deduce the estimation of IF after the *N*th iteration.

(20) $${\hat{\omega }}_{f}^{\left[N\right]}(t,\eta )=\Phi^{\prime }(t)+{\left[\frac{{\left(\Phi^{\prime \prime }(t)\right)}^{2}}{1+{\left(\Phi^{\prime \prime }(t)\right)}^{2}}\right]}^{N}\left(\eta -\Phi^{\prime }(t)\right)$$

Therefore, MSST is expressed as:

(21) $$Ts^{\left[N\right]}(t,\omega )=\frac{1}{g^{*}\left(0\right)}\int_{\left\{\eta ,\left|V_{f}^{g}(t,\eta )\right|>\gamma \right\}}^{\left(t,\eta \right)\to \left(t,{\hat{\omega }}_{f}^{\left[N\right]}(t,\eta )\right)}V_{f}^{g}(t,\eta )\delta (\omega -{\hat{\omega }}_{f}^{\left[N\right]}(t,\eta ))d\eta$$

**Figure 2:**
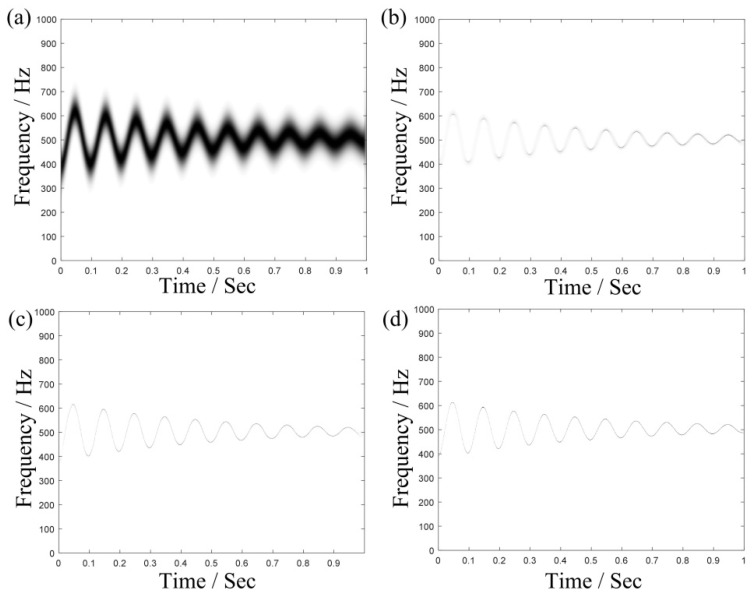
TFRs provided using (**a**) STFT; (**b**) synchrosqueezed transform (SST); (**c**) 4-SST; and (**d**) multi-synchrosqueeezing transform (MSST) (N=6).

To illustrate the advantages of MSST in sharpening time-frequency domain, we take a simple time-varying signal, $s(t)=10\cos\left[2\pi (500t-2e^{-2t}\sin(20\pi t))\right]$ as an example, and the sampling frequency of this signal is set as 4096 Hz. Figure 2 shows its TFRs obtained by SST, 2-SST, 4-SST and MSST (N=6), respectively. And their resolution is quantified by Rényi entropy shown in Table 1. In general, the smaller the value of Renyi entropy [34], the higher the resolution obtained. It proved that MSST method has the smallest value and produces more energy-intensive TFRs as shown in Table 1. 

In order to obtain stable and high resolution TFR, we directly improve MSST by referring to the idea of demodulation, so that the final TFR is a series of lines parallel to the frequency axis.

Suppose we obtained an arbitrary instantaneous frequency $f_{IF}(t)$ using TDSTFT and then the proportional coefficient at any time can be expressed as:

(22) $$K=\frac{f_{IF}(i)}{f_{IF}(t)}$$

where, $f_{IF}(i)$ represents the frequency value at the time i. Generally, *f_IF_*(*i*) is chosen among the four frequencies: initial frequency, cutoff frequency, maximum frequency, and minimum frequency, i.e., $f_{IF}(i)=f_{IF}(0)$, $f_{IF}(i)=f_{IF}(end)$, $f_{IF}(i)=f_{IF\max}(t)$, $f_{IF}(i)=f_{IF\min}(t)$.

According to the proportional coefficient K, the transformed IF is written as:

(23) $${\tilde{\omega }}_{f}^{\left[N\right]}(t,\omega )={\hat{\omega }}_{f}^{\left[N\right]}(t,K\times \omega )$$

According to (22), the IFs are redistributed on the time-frequency plane to obtain the target time-frequency representation. 

Finally, the expression of IMSST is written as follows:

(24) $$ITs^{\left[N\right]}(t,\omega )=\frac{1}{g^{*}\left(0\right)}\int_{\left\{\eta ,\left|V_{f}^{g}(t,\eta )\right|>\gamma \right\}}^{\left(t,\eta \right)\to \left(t,{\tilde{\omega }}_{f}^{\left[N\right]}(t,\eta )\right)}V_{f}^{g}(t,\eta )\delta (\omega -{\tilde{\omega }}_{f}^{\left[N\right]}(t,\eta ))d\eta$$

### 2.3. Two-Dimensional Energy-Frequency Map Obtained by IMSST

In the fault diagnosis of time-varying signals, scholars not only expect to retain as much original information as possible, but also hope to be able to produce familiar analysis graphs to directly judge, such as the Fourier spectrum, order spectrum, and so on. We can obtain high resolution time-frequency plane using IMSST. In order to further clarify the proportional relationship between signal components, it is necessary to eliminate the influence of time and transform the TFR into a 2D frequency-energy map.

The specific conversion is as follows:

(25) $$ITsre(\omega_{j})=\frac{\sum_{j=1}^{M}\left|ITs^{\left[N\right]}(t_{j},\omega )\right|}{M}$$

where, $ITsre(\omega_{j})$ represents the amplitude (energy) at frequency $\omega_{j}$ and M is the number of samples, i.e., the length of the original time series. By (25), the influence of *t* is eliminated and the corresponding 2D energy-frequency map is obtained.

**Figure 3:**
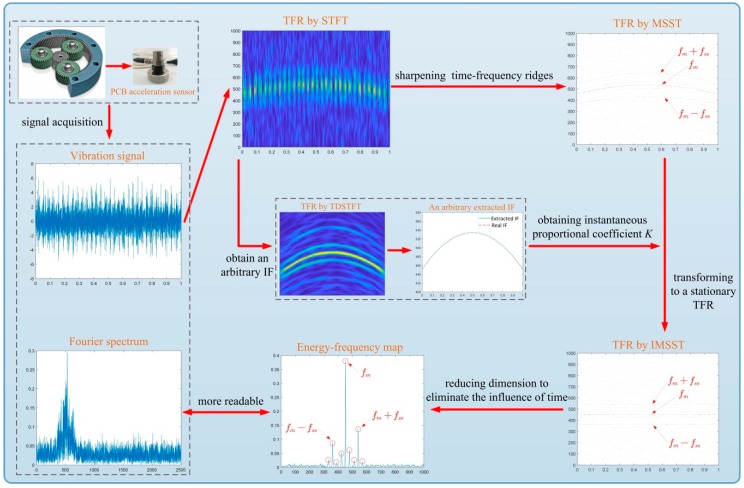
Flowchart of the proposed method.

The flowchart of the proposed method is shown in Figure 3, which is also summarized as follows:

1. Apply acceleration sensor to obtain the vibration signal s(t).
2. Extract an arbitrary IF $f_{IF}(t)$ from s(t) using TDSTFT.
3. Calculate the proportionality coefficient K according to $f_{IF}(t)$.
4. Obtain the high resolution of TFR $Ts^{\left[N\right]}(t,\omega )$ using MSST.
5. Transforming instantaneous frequency curves to a series of lines which are parallel to the frequency axis according to the proportionality coefficient K using IMSST, and then obtaining reconstructed TFR $ITs^{\left[N\right]}(t,\omega )$.
6. Eliminate the influence of time t to obtain $ITsre(\omega_{j})$ which is a result of dimensionality reduction comparing with $ITs^{\left[N\right]}(t,\omega )$, and then derive the 2D energy-frequency map.
7. Identify the fault pattern using the 2D energy-frequency map.

## 3. Simulation Validation

In this section, we describe a multicomponent signal and a planetary gear simulation signal which are employed to illustrate the effectiveness of the proposed method.

### 3.1. Multicomponent Signal

**Figure 4:**
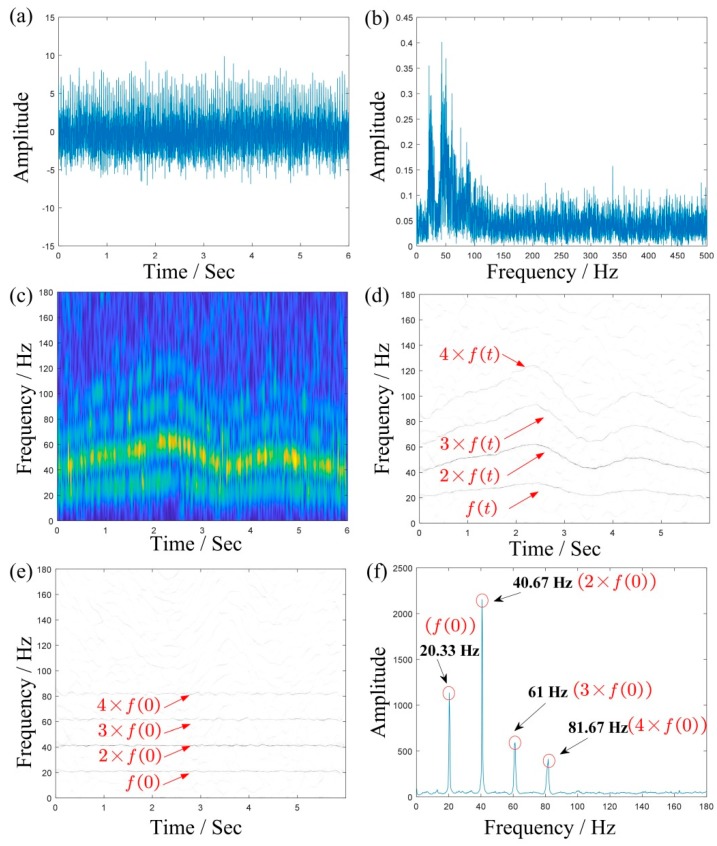
The result of a multicomponent simulated signal: (**a**,**b**) are the waveform and the Fourier spectrum; (**c**–**e**) are the TFRs obtained using STFT, MSST, and IMSST, respectively; and (**f**) is the 2D energy-frequency map.

To better illustrate the principle of this method, a simple time-varying multicomponent signal is established in this section, which consists of four sinusoidal functions with proportional frequencies.

(26) $$\{\begin{array}{l} s(t)=\sum_{k=1}^{4}A_{k}\cos\left(2\pi \int_{0}^{t}k\times f(t)dt\right)+n(t)\\ f(t)=12\sin\left(0.35t+\frac{\pi }{4}\right)+3\sin\left(2t-\frac{4\pi }{5}\right)+1.62\sin\left(3.5t-\frac{7\pi }{3}\right)+50 \end{array}$$

where, f(t) is the IF and n(t) is the white Gaussian noise. In this signal, the sampling frequency is 1000 Hz and the sampling number is 6000. Similarly, a SNR of −5 dB is added to form a synthetic signal, as shown in Figure 4a. The Fourier spectrum of the synthetic signal is shown in Figure 4b. For the influence of variable speed, the energy of Fourier spectrum is divergent and blurry, which means the useful information is hard to find.

For comparison, the TFR results of the signal obtained using STFT, MSST, and IMSST are presented in Figure 4c–e. Due to the influence of the Heisenberg uncertainty principle [17], the TFR of STFT has the characteristics of a large frequency bandwidth and a low frequency resolution. Especially in noise background, the whole time-frequency plane is basically blurred, which corresponds to the result in Figure 4c. Figure 4d,e are TFRs obtained using MSST and IMSST, respectively. In comparison to Figure 4c, Figure 4d and e shows sharper time-frequency ridges. Moreover, Figure 4e transforms the time-varying instantaneous frequency ridge into a line parallel to the frequency axis on the basis of retaining the high resolution of Figure 4d, which makes the proportional relationship between the components clearer. Figure 4f is a 2D energy-frequency map derived from the TFR of Figure 4e which shows the four proportional frequencies: 20.33 Hz, 40.67 Hz, 61 Hz, and 81.67 Hz, which correspond to the integral times of f(0)=20.32 Hz basically. There is no doubt that Figure 4f is more readable than Figure 4e.

**Figure 5:**
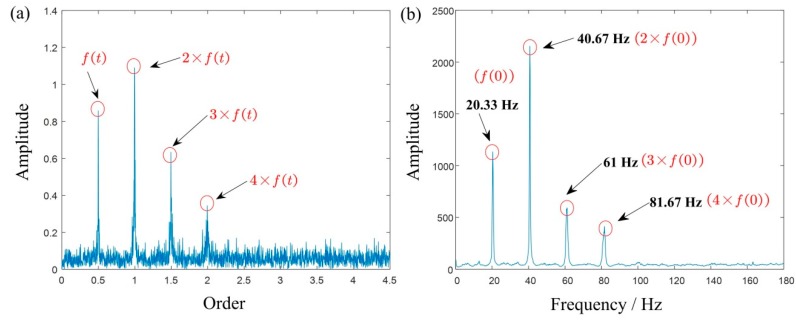
The result analysis of a multicomponent simulated signal: (**a**) order spectrum and (**b**) 2D energy-frequency map.

For further comparison, we draw the final results of the OT method and the proposed method in Figure 5. It should be noted that the result of the OT method is obtained through three steps: firstly, obtain an IF using the peak searching algorithm on the TFR by STFT. Secondly, apply the angle domain resampling technology to obtain the stable angle domain signal. Thirdly, draw the Fourier spectrum of the angle domain signal. As compared with the results of OT and the proposed method, we see that the latter not only highlights the feature components, but also reduces the influence of noise, which indicates that the proposed method produces clearer results.

### 3.2. The Simulated Planetary Gear Signal under Time-Varying Rotating Speed

To evaluate the effectiveness of the proposed method for analyzing planetary gear vibration signals, the simulated planetary gear signals are generated according to [35]:

(27) $$\begin{array}{c} s(t)=\left[1-\cos\left(2\pi \int f_{s}^{\left(r\right)}(t)dt\right)\right]\left[1+A_{1}\cos\left(2\pi \int f_{ss}(t)dt\right)+\theta_{1}\right]\times \\ \;\cos\left[2\pi \int f_{m}(t)dt+A_{2}\sin\left(\int f_{ss}(t)dt+\theta_{2}\right)+\theta_{3}\right]+n(t) \end{array}$$

where, $f_{s}^{\left(r\right)}(t)$ is the instantaneous rotational frequency of sun gear and $f_{ss}(t)$ is the instantaneous fault characteristic frequency (IFCF). $f_{m}(t)$ denotes the instantaneous meshing frequency of the simulated signal. $A_{1}$ and $A_{2}$ are expressed as modulation factors. $\theta_{1}$, $\theta_{2}$ and $\theta_{3}$ are initial phases. n(t) corresponds to the while Gaussian noise. All parameters are shown in Table 2.

Considering the variable speed, the instantaneous rotation frequency $f_{s}^{\left(r\right)}(t)$ is written as:

(28) $$f_{s}^{\left(r\right)}(t)=-20t^{2}+20t+27$$

**Figure 6:**
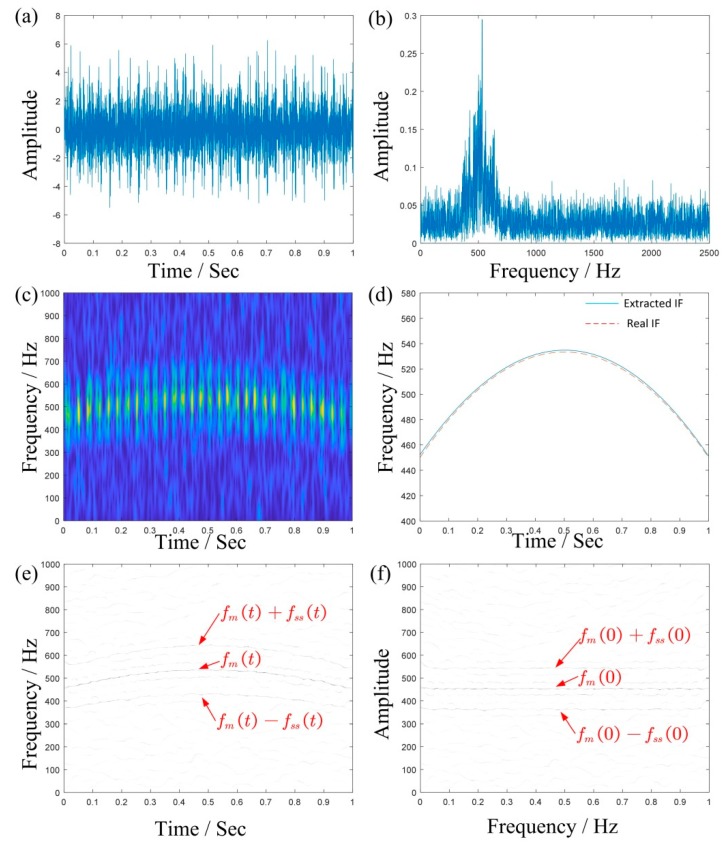
The result of simulated signal: (**a**,**b**) are the waveform and the Fourier spectrum; (**d**) is the extracted IF by TDSTFT, where the blue line is the extracted IF and the red line is the real IF; (**c,e**,**f**) are the TFRs obtained using STFT, MSST, and IMSST, respectively.

Figure 6a,b shows the time domain waveforms and the Fourier spectrum of the simulated signals, respectively. The TFR obtained using STFT is shown in Figure 6c, where we can roughly see that the energy is concentrated between 400 and 600 Hz. However, it is difficult to further distinguish the meshing frequency and its sideband frequency. Figure 6d is the extracted IF by TDSTFT, which is basically close to the true value (where the blue line is the extracted IF and the red line is the real IF). Figure 6e,f are TFRs obtained using MSST and IMSST, respectively. As comparing with Figure 6d, they are clearer to find the meshing frequency and its sideband frequency.

**Figure 7:**
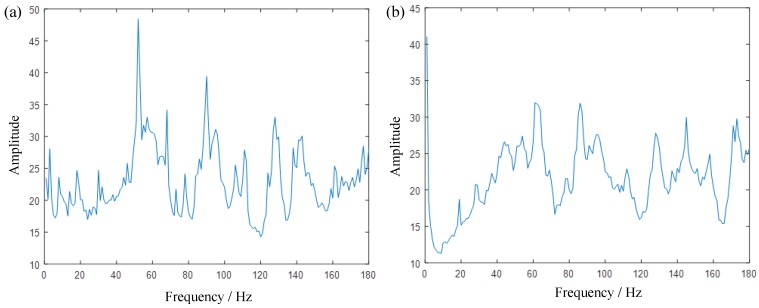
(**a**,**b**) are the 2D energy-frequency maps by SST and 4-SST based on the same algorithmic theory in Section 2.2 and Section 2.3, respectively.

**Figure 8:**
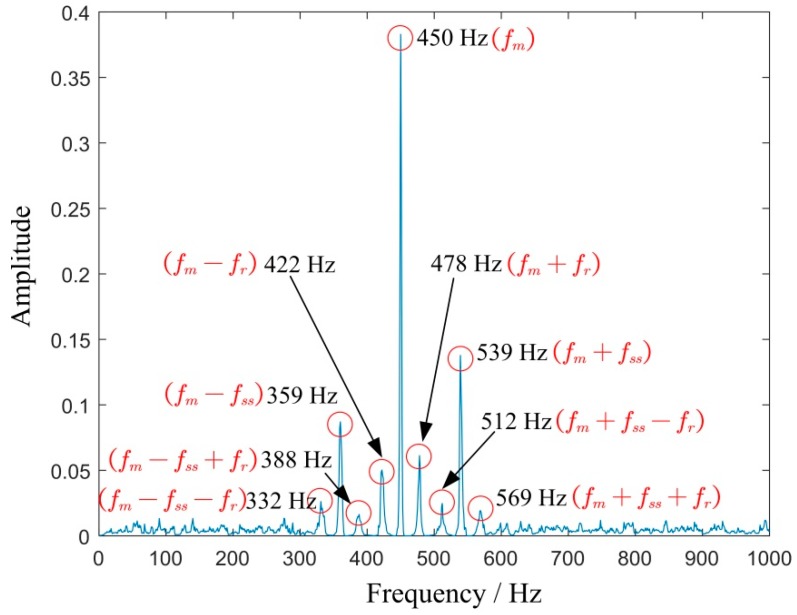
2D energy-frequency map by proposed method.

To better illustrate the advantage of the method, we respectively generate the 2D energy-frequency maps by SST and 4-SST base on the same algorithmic theory in Section 2.2 and Section 2.3, in Figure 7. Due to the aliasing of each component, it is difficult to capture useful peaks from the graph. This is mainly because two methods cannot provide enough clear TFRs, and therefore it results in blurred 2D energy-frequency maps. Figure 8 is the 2D energy-frequency map using IMSST (N=20) of a planetary gear simulated signal, which clearly shows the meshing frequency and its corresponding sideband. In comparison to Figure 6b, the phenomenon of frequency divergence has been greatly improved. Furthermore, the proportional relationship among the components exhibits more clearly in the energy-frequency map as comparing with those in Figure 7. Moreover, the results perfectly realize the transformation from non-stationary signal to stationary signal, which is also convenient for fault diagnosis.

**Figure 9:**
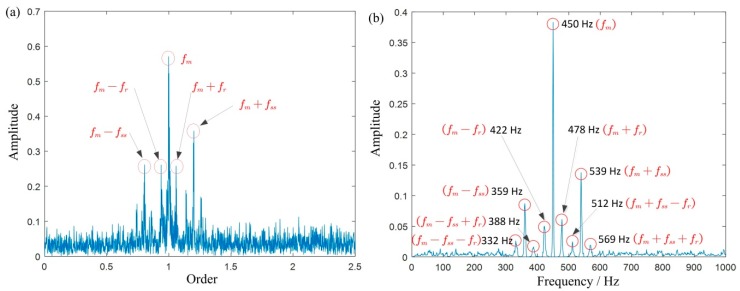
The result analysis of a multicomponent simulated signal: (**a**) order spectrum and (**b**) 2D energy-frequency map.

For further comparison, we also draw the results of the OT method and the proposed method in Figure 9 which shows that the proposed method is better for highlighting fault characteristic frequency and eliminating background noise.

**Figure 10:**
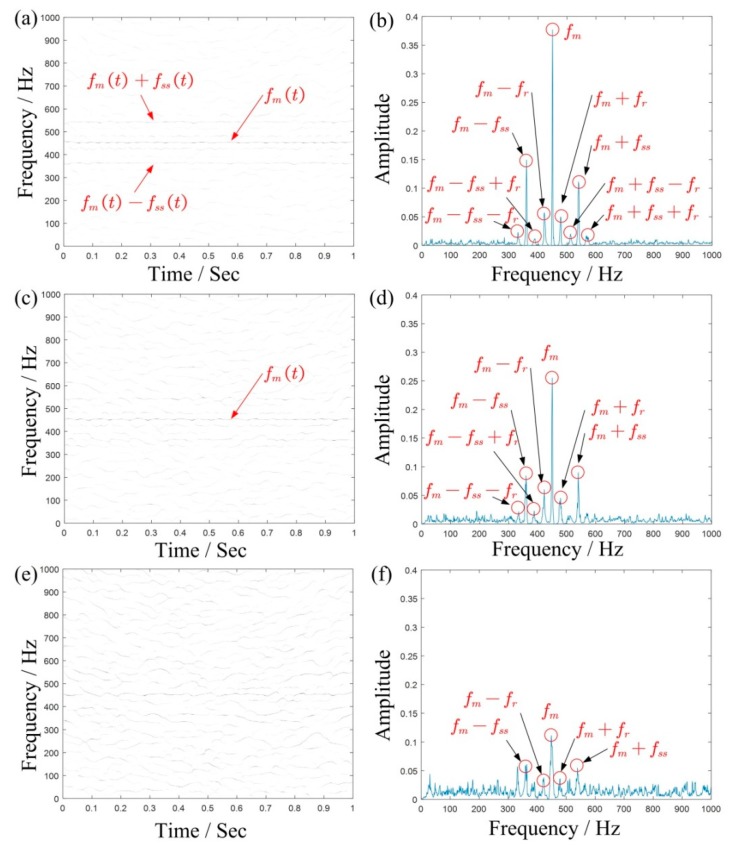
The result analysis of simulated signals under different noise levels: (**a**,**c**,**e**) are TFRs using IMSST, respectively; (**b**,**d**,**f**) are the 2D energy-frequency maps, respectively.

To further testify the robustness of the proposed method, more simulated signals under different noise levels are analyzed. Figure 10 presents the TFRs using IMSST and the corresponding 2D energy-frequency maps of the simulated signals with SNR of 0 dB, −5 dB, and −10 dB, respectively. With the enhancement of noise, it becomes more and more difficult to directly recognize the interest components from TFR using IMSST. Correspondingly, the peak value of characteristic frequencies becomes less prominent in the 2D energy-frequency maps. Therefore, when the noise SNR of a simulated signal is greater than −5 dB, the proposed method performs well with respect to fault feature recognition. If the noise level is too high, the effectiveness of the proposed method is not ideal. It is worth noting that, when the signal noise is very strong, it can be combined with the existing singular spectrum analysis (SSA), convex optimization, time-varying filter, and other denoising methods to reduce the noise level of the signal.

## 4. Experimental Validation

As we all know, the simulated signal is usually a vibration model based on the meshing characteristics of gearbox which is a simplified AM-FM model. The real acquisition signal contains not only the coupling vibration information of key components such as gears, bearings, and so on, but also the other interference information caused by background noise and vibration of other components of the mechanical equipment. It is more complex than the simulation signal. Therefore, it is necessary to verify the proposed method by experiment.

### 4.1. Experimental Rig

**Figure 11:**
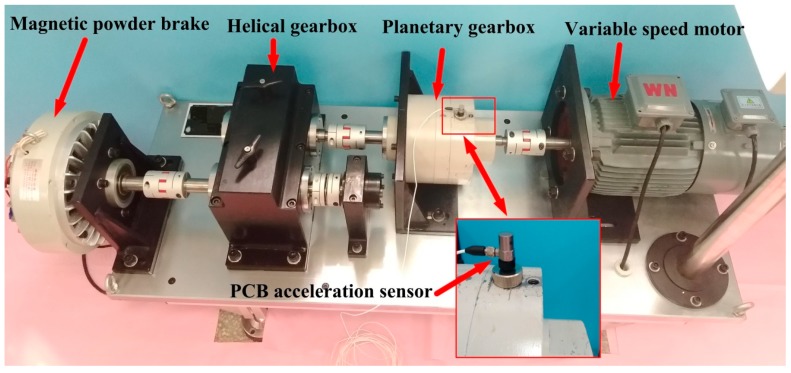
The experimental rig.

**Figure 12:**
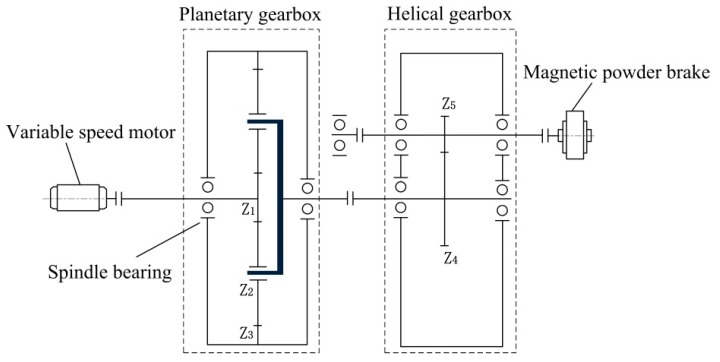
The structure of experimental rig.

**Figure 13:**
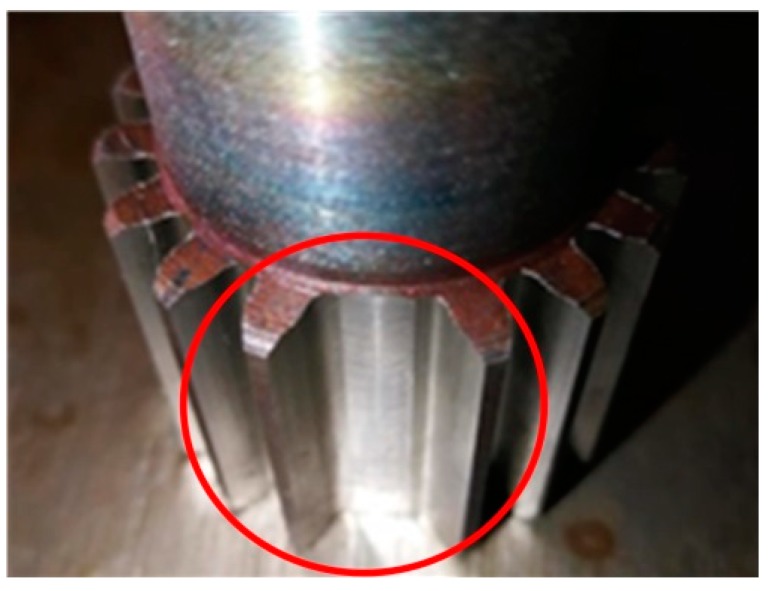
The damage of the sun gear.

In this section, the proposed method is applied to detect the gear damage. The experiment is carried out on the test rig shown in Figure 11. The experimental rig is composed of a driving motor, planetary gear box, helical gearbox, magnetic powder brake, and other components. The internal structure of the test rig is shown in Figure 12. The test fault is located on the sun gear of planetary gearbox, as shown in Figure 13. A PCB accelerometer is mounted on the top of the planetary gearbox to measure the vibration signal.

In this experiment, the rotational speed of the motor decreases linearly from 3000 r/min with a sampling frequency of 2560 Hz and a sampling number of 7680. The applied torque by the magnetic powder brake is 0. The parameters of the planetary gearbox are shown in Table 3, and the corresponding fault characteristic frequencies are calculated with reference to [36], as shown in Table 4.

### 4.2. Result Analysis

**Figure 14:**
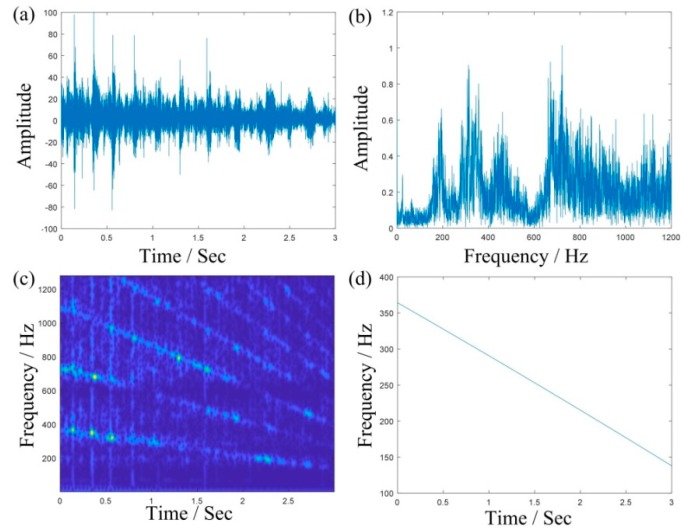
Vibration signal acquired from the planetary gearbox. (**a**) waveform; (**b**) Fourier spectrum, (**c**) TFR by STFT, and (**d**) the extracted IF by TDSTFT.

Figure 14a,b are the time domain waveform and the Fourier spectrum of the fault vibration signals of the sun gear, respectively. As shown in Figure 14a, the impact decreases with a decrease of rotational speed. Due to the influence of variable speed, it is still difficult to identify the fault type directly from the Fourier spectrum. Figure 14c shows the TFR using STFT, where we can roughly see the changing trend of the vibration signal. $f_{IF}(t)$ is the IF extracted using TDSTFT (see Figure 14d), which shows that the speed of the vibration signal basically decreases in a linear way, which is also in line with our Introduction.

According to the extracted IF, select $f_{IF}(0)$ as the molecule of the proportional coefficient *K*, and then we obtain $K=\frac{f_{IF}(0)}{f_{IF}(t)}$. At this time, the variable speed signal will be converted to a constant frequency according to the proportional relationship. Then the rotation frequency of the sun gear in the energy-frequency map is $f_{s}^{\left(r\right)}=23.5$ Hz, and the other frequencies are calculated according to Table 4.

**Figure 15:**
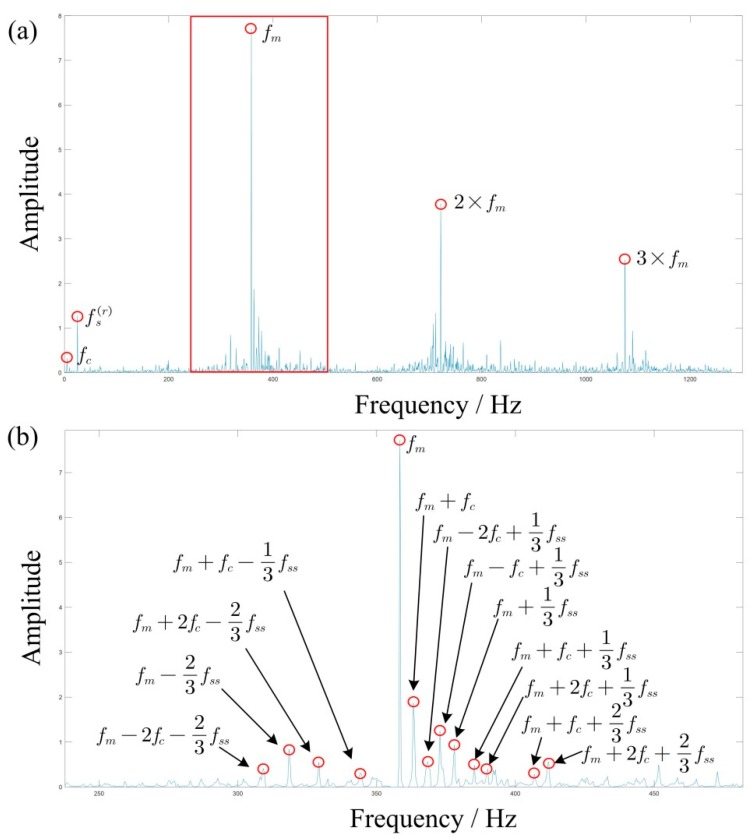
(**a**) 2D energy-frequency map of the vibration signal and (**b**) an enlarged view.

Generally, if the three planetary gears meshing with the sun gear are identical, the FCF of the sun gear is expressed as $f_{ss}=Nf_{m}/Z_{s}$, where N is the number of planetary gears and $Z_{s}$ is the number of sun gear teeth. And the peak value of sideband usually appears at the frequency of $f_{m}\pm kf_{s}^{\left(r\right)}+nf_{ss}$. On the basis of the equation $f_{ss}=N\left(f_{s}^{\left(r\right)}-f_{c}\right)$, the above frequency can also be written as $f_{m}\pm kf_{c}+\left(n\pm k/N\right)f_{ss}$. However, manufacturing error is inevitable in the actual gearbox, which means the three planetary gears meshing with the sun gear cannot be exactly identical, leading to the difference of the impact sequence between the fault teeth of the sun gear and three planetary gears. If these impacts are considered as three different sequences, the FCF of the sun gear can be written as $f_{ss}/N$. Correspondingly, the peak value of the sideband appears at frequency $f_{m}\pm kf_{c}+\left((n\pm k)/N\right)f_{ss}$. Figure 15a is the final 2D energy-frequency map, where the rotational frequency $f_{s}^{\left(r\right)}$ of the sun gear; the rotational frequency $f_{c}$ of the planetary frame; and the meshing frequency, $f_{m}$ and $2f_{m}$, $3f_{m}$ are easily found. Figure 15b is an enlarged view of Figure 13 near the frequency *f_m_* and shows that the corresponding peak values are related to one-third times the characteristic frequency of the sun gear fault characteristic frequency, which could be caused by the manufacturing or installation errors of the three planetary gears. However, this does not prevent us from judging that the fault of the planetary gearbox is the broken teeth of sun gear.

## 5. Conclusions

In this paper, a novel fault diagnosis method for time-varying signals was proposed, and the performance of this method has been verified using simulation signals and a planetary gearbox experiment. Its advantages are as follows:

Firstly, this method does not need to estimate the instantaneous shaft frequency. It only needs to estimate an arbitrary instantaneous frequency with time-varying characteristics, such as gear meshing frequency, which is easier than extracting the instantaneous shaft frequency.

Secondly, this method can smooth the time-varying frequency ridges and achieve the effect similar to GD and OT. Moreover, this method omitted the steps of filtering and resampling, so that it can retain the complete information of the vibration signal.

Finally, this method can generate a 2D energy-frequency map, which is similar to the spectrum map. In addition, in comparison to direct analysis on time-frequency plane, this form is more intuitive and friendly.

## Figures and Tables

**Table 1:** Renyi entropy values of TFRs.

TFA	STFT	SST	4-SST	MSST (N=6)
Renyi entropy	19.1162	15.3511	13.5911	12.0610

**Table 2:** Parameters of simulated signal.

$f_{s}^{\left(r\right)}(t)$	$f_{m}(t)$	$A_{1}$	$A_{2}$	$\theta_{1}$	$\theta_{2}$	$\theta_{3}$	n(t)
$\left(10/3\right)f_{s}^{\left(r\right)}(t)$	$\left(50/3\right)f_{s}^{\left(r\right)}(t)$	1	0.05	0	0	0	−1 dB

**Table 3:** Configuration of the planetary gearbox.

Gear	Sun Gear	Planet Gear	Ring Gear
Number of Gear teeth	18	27 (3)	72

**Table 4:** Fault characteristic frequency of planetary gearbox.

Gear Meshing Frequency	Absolute Rotating Frequency		Fault Characteristic Frequency		
	Sun Gear	Planet Carrier	Sun Gear	Planet Gear	Ring Gear
$f_{m}=14.4f_{s}^{\left(r\right)}$	$f_{s}^{\left(r\right)}$	$f_{c}=0.2f_{s}^{\left(r\right)}$	$f_{ss}=2.4f_{s}^{\left(r\right)}$	$0.53f_{s}^{\left(r\right)}$	$0.2f_{s}^{\left(r\right)}$
